# Systematic selection and validation of suitable reference genes for quantitative real-time PCR normalization studies of gene expression in *Nitraria tangutorum*

**DOI:** 10.1038/s41598-020-73059-3

**Published:** 2020-09-28

**Authors:** Bo Wang, Huirong Duan, Peifang Chong, Shiping Su, Lishan Shan, Dan Yi, Lirong Wang, Yi Li

**Affiliations:** 1 College of Forestry, Gansu Agricultural University, Lanzhou, 730070 China; 2 The College of Ecological Environment and Resources; Institute of Ecology and Environment of Qinghai-Tibet Plateau; Key Laboratory of Biotechnology and Analysis and Test in Qinghai-Tibet Plateau; Laboratory of Resource Chemistry and Eco-Environmental Protection in Tibetan Plateau, Qinghai Nationalities University, Xining, 810007 China; 3 Lanzhou Institute of Husbandry and Pharmaceutical Science, Chinese Academy of Agricultural Sciences, Lanzhou, 730000 China

**Keywords:** Molecular biology, Plant sciences

## Abstract

Suitable reference genes can be used to calibrate the error in quantitative real-time PCR (qPCR) experiments, making the results more credible. However, there are no reference genes suitable for multiple species and under different experimental conditions. *Nitraria tangutorum* Bobr. is a typical plant native to desert areas. It is drought-resistant, saline-alkali resistant, extreme temperatures-resistant, and has strong adaptability. To date, the importance of this germplasm has not been sufficiently understood; therefore, it is still unclear which genes can be used as reference genes to calibrate qPCR data of *N*. *tangutorum.* In this study we analyzed the expression levels of 10 candidate reference genes (*ACT*, *GAPDH*, *TUA*, *TUB*, *CYP*, *UBC*, *His*, *PP2A*, *HSP*, and *EF1-α*) in *N. tangutorum* seedlings under a series of experimental conditions, including in different organs (root, stem, and leaf) and under abiotic stresses (salt, drought, heat, and cold) and hormone stimuli (abscisic acid) by qPCR. Three software programs (geNorm, NormFinder, and BestKeeper) were used to evaluate the expression stability of the ten genes. Comprehensive analysis showed that *EF1-α* and *His* had the best expression stability, whereas *HSP* was the least suitable as a reference gene*.* The expression profile of *NtCER7*, a gene related to the regulation of cuticular wax biosynthesis in *N*. *tangutorum*, verified the accuracy of the experimental results. Based on this study, we recommend *EF1-α* and *His* as suitable reference genes for *N*. *tangutorum*. This paper provides the first data on stable reference genes in *N*. *tangutorum*, which will be beneficial to studying the gene expression of *N*. *tangutorum* and other *Nitraria* species in the future.

## Introduction

Gene expression research has become an important means of revealing gene function and molecular mechanisms. Quantitative real-time PCR (qPCR), a method that can accurately analyze gene expression, is favored by most scientific researchers^1−3^. An appropriate reference gene can effectively reduce the error in the qPCR experimental process, resulting in more credible results^4^. Housekeeping genes, which have a stable expression in cells and help maintain cell functions, are generally used as reference genes. The expression level of ideal reference genes in plants does not change significantly with changes in tissue locations, life cycle stages, and external environmental conditions^5−7^. However, research has revealed that reference genes do not have universal applicability. That is, reference genes that are stably expressed in different species and under different experimental conditions do not exist^8−10^. Therefore, selecting suitable reference genes is the key to analyzing the gene expression level in plants under specific conditions by qPCR.

Reference genes are often used to calibrate the results of qPCR, so a stable reference gene is required for qPCR. The commonly used reference genes are those that constitute the cytoskeleton or are involved in the basic metabolic activities of cells, for example, *ACT*, *GAPDH*, *EF1-α*, *TUA*, *TUB*, and *His.* Most previous studies on selected suitable reference genes focused on model plants^11−15^. However, with our increasing understanding of the agricultural ecological value of non-model plants, it has become important to study their molecular genetic mechanisms and to determine their appropriate reference genes under different experimental conditions. To date, some non-model plants have been selected for research on internal reference genes, including *Reaumuria*^16^, *Caragana intermedia*^17^, *Setaria viridis*^18^, *Miscanthus lutarioriparia*^19^, *Haloxylon ammodendron*^20^, *Hylocereus undulatus Britt*^21^, and *Corchorus capsularis *L^22^.

*Nitraria tangutorum* Bobr., belonging to the Zygophyllaceae family, is mainly found in the arid, salinized desert of northwest China. Its adaptability to the extreme environmental conditions of the desert allow it to survive in harsh habitats^23^. In recent years, reports on *N. tangutorum* have mainly focused on the physiological mechanisms of its drought and salt tolerance^24−27^, which indicate that this species has very important ecological functions. With the development of molecular biology, gene expression analysis may help reveal the molecular mechanism of stress responses in *N. tangutorum*. Screening for suitable *N. tangutorum* reference genes under different experimental conditions is therefore of great significance. Unfortunately, there have been no reports in this area so far. In this study, based on our previous transcriptome analysis, 10 commonly used reference genes (*ACT*, *UBC*, *TUA*, *TUB*, *GAPDH*, *CYP*, *PP2A*, *His*, *HSP*, and *EF1-α*) in *N. tangutorum* were cloned. Their expression levels under a series of experimental conditions, including in different organs (root, stem, and leaf), under various abiotic stresses (salt, drought, heat, and cold), and under hormone stimuli [abscisic acid (ABA)] were detected by qPCR. Three different software packages (geNorm^29^, Normfinder^30^, and BestKeeper^31^) were used to evaluate the expression stability of the ten genes. In order to further verify the reliability of the reference gene selection results, we analyzed the expression pattern of *NtCER7*, a gene related to the regulation of the cuticular wax biosynthesis of *N. tangutorum* under salt stress and in different organs. These results provide a theoretical basis for subsequent research on the regulation of functional gene expression in *N. tangutorum*.

## Results

### Selection of reference genes and verification of primer specificity

We selected 10 genes as candidate genes by mining the *N. tangutorum* transcriptome data according to previous reports on the selection of reference genes in model plants and non-model plants^13–22^. The chosen *N. tangutorum* genes were then used to query against the *Arabidopsis* protein database using BLASTX to obtain the homologous genes. The annotation and comparison information of the 10 reference genes are listed in Supplementary Table S1. Based on the unigene sequences, qPCR primers were designed. The amplification efficiency of these 10 pairs of primers ranged from 93.12 to 109.07%, and the correlation coefficients (*R*^2^) ranged from 0.9762 to 0.9998 (Table 1). The melting curve only had a single melting peak, and there was no non-specific amplification (see Supplementary Fig. S1). The results indicated that the primers of these 10 genes were reasonably designed and had good specificity, which met the qPCR standards and can be used for subsequent experiments.

**Table 1:** Details of primers used in this study.

Genes	Primer sequence forward/reverse (5′–3′)	Tm (°C)	Length (bp)	Efficiency (%)	*R*^2^
*ACT*	TCGTGTTGCCCCTGAAGAACACCCCGT	68.8	129	107.14	0.9911
	/TGGATGGCGACGTACATAGCGGGCA	67.3			
*GAPDH*	ACCCTGAGGAGATCCCATGGGGTGA	66.34	107	97.45	0.9998
	/TGCACCACCCTTCAAGTGAGCAGCA	65.79			
*TUA*	ACCAGTGCCTCCACCAACAGCA	64.55	145	101.6	0.9944
	/TGCCGCCAATAACTTTGCCAGAGGA	63.48			
*TUB*	AGCTCAGGAACAGTGAGGGCCCTGT	67.05	155	101.89	0.9762
	/TGCTGCCTTCGATTCCCTGGTCA	63.64			
*CYP*	TGGGCCAAATACGAACGGGTCCCA	65.82	162	105.56	0.9975
	/ACAACAGGCTGCGAAGTCCTCCCA	65.67			
*UBC*	ACCCACCAACTTCATGCAGTGCAGGT	65.47	106	98.72	0.9815
	/ACGAAAACACCTCCGGCGTAGGGGCTA	68.2			
*His*	AGGAGGCGTCGAGATTGGCGAGGTA	66.5	123	93.12	0.9977
	/TGGTCCCCTCAGAAACAGCGTGCT	65.67			
*PP2A*	TGGACAGGCAGATCGCGCACTT	64.55	127	109.07	0.9861
	/ACCGGACACTTGACTGGCTGCACGT	67.77			
*HSP*	TTGTCTGCCTCGGCTCTCTTCCGCA	67.25	177	102.4	0.9902
	/TCCTTGCTGCTTGGTGACCGGA	64.23			
*EF1-α*	ACGCTCACGCTCGGCCTTAAGCTT	66.03	176	97.9	0.9855
	/TGGTCATTGGCCACGTCGACTCTGG	65.99			
*NtCER7*	ACCCGACTCACCACGAGGAAGCTGT	66.87	149	96.66	0.9885
	/GGTCCTTGACGCAAGCCGCAAGCAT	67.41			

### Analysis of the Cq value of reference genes

**Figure 1:**
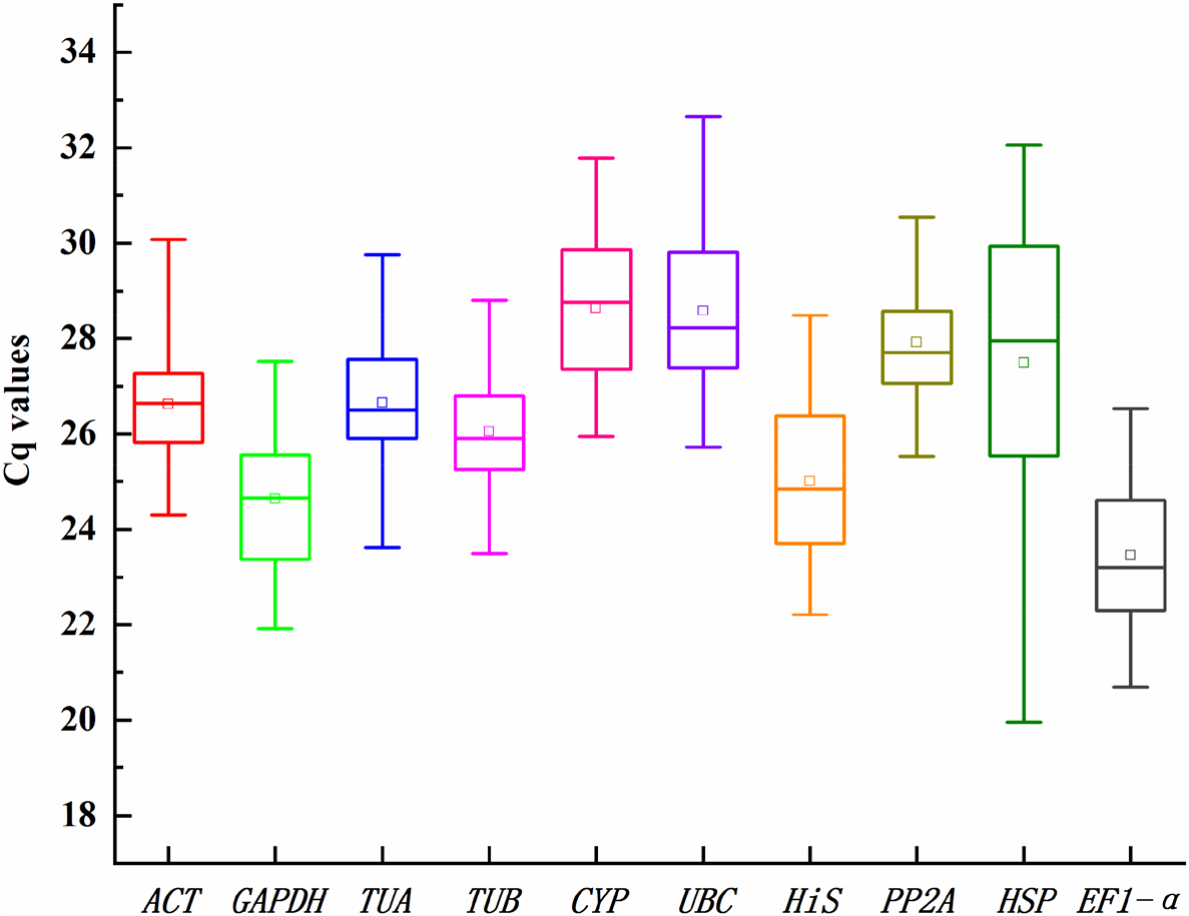
Cq values of 10 candidate reference genes in all *N. tangutorum* samples. The block diagram shows the quartile range. The outer box indicates the 25th to 75th percentile, and the inner box indicates the average. The horizontal lines inside the box are the median lines. Whiskers indicate the minimum and maximum values. The figures were generated by using OriginPro software (version 2018; https://www.originlab.com/).

The Cq value is inversely proportional to the transcription level of a gene, that is, higher Cq values correspond to lower expression abundance. A change in the Cq value of the same gene in different samples reflects the variation of the expression level. In this study, the Cq values of the 10 genes changed under a series of experimental conditions, including in different organs (root, stem, and leaf), and under various abiotic stresses (salt, drought, heat, and cold) and hormone stimuli (ABA). The change trends were found to be different (Fig. 1). Among the 10 reference genes, the lowest and highest Cq values were found in *HSP* and *UBC* at 19.96 and 32.65, respectively, indicating a wide range of expression abundance. The range of Cq values of *ACT*, *GAPDH*, *TUA*, *TUB*, *CYP*, *UBC*, *His*, *PP2A*, *HSP*, and *EF1-α* ranged from 24.30–30.08, 21.92–27.53, 23.62–29.77, 23.50–28.80, 25.50–31.79, 25.72–32.65, 22.21–28.48, 25.54–30.54, 19.96–32.06, and 20.70–26.54, respectively (Supplementary Table S2). Among the 10 genes, the expression abundances of *EF1-α*, *GAPDH*, *His*, *ACT*, *TUB*, and *TUA* had the highest expression abundances and the smallest range of variation. *CYP*, *UBC*, and *PP2A* had low expression abundance and low variation in expression levels. The expression level of *HSP* was the most variable. Our preliminary results indicated that the expression levels of *EF1-α*, *GAPDH*, *His*, *ACT*, *TUB*, and *TUA* were relatively stable, and the expression of *HSP* varied greatly. For greater accuracy, these results need to be evaluated by reference gene analysis software.

### GeNorm analysis

**Figure 2:**
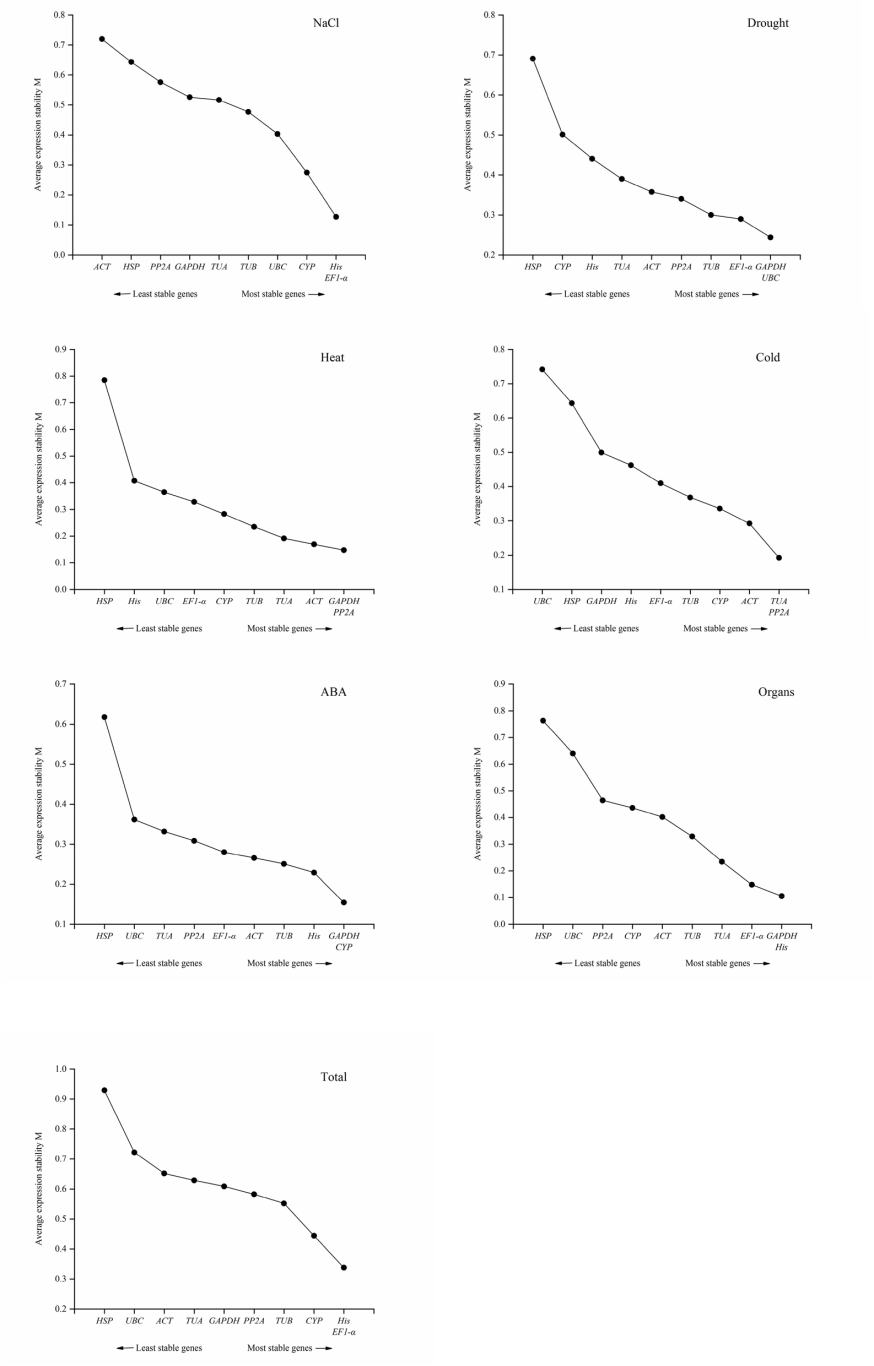
Average expression stability values (M) of the 10 candidate reference genes based on the geNorm algorithm. The lower the M value, the more stable the expression. The most stable genes are to the right and the most unstable genes are on the left. The figures were generated by using OriginPro software (version 2018; https://www.originlab.com/).

Using geNorm analysis, we obtained the stable expression M value of the reference gene, which is negatively correlated with the stability of the gene. That is, a larger M value represents a more unstable gene. When all the samples were combined, the M values of *His* and *EF1-α* were the lowest and that of *HSP* was the highest, indicating that the expression levels of *His* and *EF1-α* were the most stable, and the expression of *HSP* was the least stable. For stable expression levels under salt, drought, heat, cold, and ABA stress, and among different organs, the top two genes were *His* and *EF1-α*, *GAPDH* and *UBC*, *GAPDH* and *PP2A*, *TUA* and *PP2A*, *GAPDH* and *CYP*, and *GAPDH* and *His*, respectively (Fig. 2).

**Figure 3:**
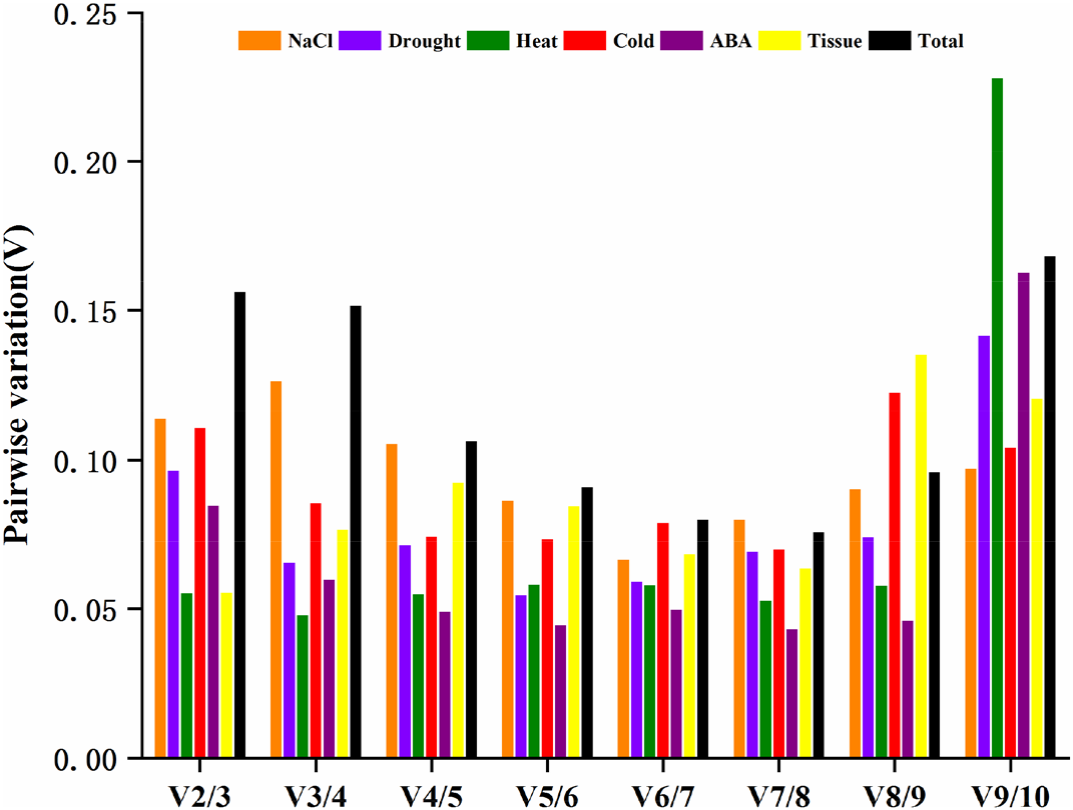
Pairwise variation (V) of the 10 candidate reference genes calculated by geNorm to determine the optimal number of reference genes for accurate normalization. The threshold used was 0.15. The figures were generated by using OriginPro software (version 2018; https://www.originlab.com/).

GeNorm can calculate the best number of genes when selecting multiple reference genes. It uses the relationship between the threshold V and 0.15 to determine whether an additional reference gene needs to be added. The principle is that when a new reference gene is introduced, the paired variation value (V value) will change. By calculating the ratio *Vn/n* + 1, we can know whether a new reference gene needs to be introduced; 0.15 is the default *Vn/n* + 1 threshold value of the software. If *Vn/n* + 1 > 0.15, the *n* + 1 reference gene needs to be introduced, otherwise it is not needed. In samples under salt, drought, heat, cold, and ABA stress, as well as in different organs, the *V2/V3* values were all less than 0.15, indicating that it was not necessary to introduce a third reference gene to calibrate the qPCR data. However, when analyzing the samples of all experimental conditions, the *V2/V3* value was 0.156, which exceeded the threshold of 0.15. The reason for this may be that the candidate reference genes of *N. tangutorum* changed significantly under different abiotic stresses (drought, salt, heat, and cold), in different organs (root, stem and leaf), and under hormone stimuli (ABA). At this time, the use of two reference genes cannot meet the needs of calibrating qPCR data (Fig. 3).

### NormFinder analysis

The NormFinder software directly evaluated the stability of reference genes according to the variance within and between groups. Generally, the smaller the stability value, the better the stability of the corresponding gene expression. As shown in Table 2, in all samples, *EF1-α*, *His*, and *GAPDH* had the lowest stability values at 0.068, 0.080, and 0.080, respectively. This showed that among the 10 reference genes, *EF1-α*, *His*, and *GAPDH* had the best stability. *HSP* had the highest stability value at 0.161, indicating that it had the highest variability. This concurred with the geNorm analysis. The highest ranked pairs of genes under different experimental conditions were not completely consistent. For example, *PP2A* and *HSP*, *UBC* and *EF1-α*, *ACT* and *PP2A*, *EF1-α* and *CYP*, *GAPDH* and *His*, and *EF1-α* and *TUA* were the top ranked genes under salt, drought, heat, cold, ABA stress, and in different organs, respectively.

**Table 2:** Expression stability of the 10 candidate reference genes calculated by NormFinder.

Rank	Salt treatment		Drought treatment		Heat treatment		Cold treatment		ABA treatment		Different organs		Total samples	
	Gene	Stability	Gene	Stability	Gene	Stability	Gene	Stability	Gene	Stability	Gene	Stability	Gene	Stability
1	*PP2A*	0.06	*UBC*	0.033	*ACT*	0.008	*EF1-α*	0.018	*GAPDH*	0.017	*EF1-α*	0.045	*EF1-α*	0.068
2	*HSP*	0.066	*EF1-α*	0.047	*PP2A*	0.01	*CYP*	0.037	*His*	0.041	*TUA*	0.053	*His*	0.08
3	*EF1-α*	0.09	*GAPDH*	0.061	*TUA*	0.024	*GAPDH*	0.07	*PP2A*	0.049	*GAPDH*	0.067	*GAPDH*	0.08
4	*CYP*	0.096	*PP2A*	0.064	*GAPDH*	0.03	*His*	0.075	*EF1-α*	0.049	*TUB*	0.073	*PP2A*	0.1
5	*GAPDH*	0.099	*His*	0.067	*CYP*	0.036	*ACT*	0.077	*CYP*	0.056	*His*	0.084	*TUB*	0.116
6	*TUB*	0.1	*TUB*	0.079	*TUB*	0.037	*UBC*	0.089	*TUB*	0.061	*UBC*	0.114	*CYP*	0.123
7	*TUA*	0.102	*ACT*	0.095	*His*	0.038	*PP2A*	0.094	*TUA*	0.095	*HSP*	0.136	*ACT*	0.132
8	*His*	0.106	*TUA*	0.142	*EF1-α*	0.039	*TUA*	0.117	*ACT*	0.112	*CYP*	0.151	*TUA*	0.136
9	*UBC*	0.108	*CYP*	0.156	*HSP*	0.133	*TUB*	0.159	*UBC*	0.115	*ACT*	0.157	*UBC*	0.139
10	*ACT*	0.151	*HSP*	0.198	*UBC*	0.139	*HSP*	0.217	*HSP*	0.14	*PP2A*	0.206	*HSP*	0.161

### BestKeeper analysis

The BestKeeper algorithm calculates the standard deviation (SD) and coefficient of variation (CV) using Excel software based on the Cq value of the gene. The smaller the SD and the smaller the CV, the better the stability of the gene. If SD > 1, the gene is considered unstable. The analysis results are shown in Table 3. In all samples, only *ACT* and *PP2A* showed SD values < 1, which met the requirements for reference genes. However, this was different from the analysis results of geNorm and NormFinder. There were differences in the number of genes that met the SD < 1 condition under different experimental conditions. Under the salt, drought, heat, cold, and ABA stress and in different organs, the highest ranked pairs of genes were *ACT* and *PP2A*, *TUA* and *ACT*, *UBC* and *CYP*, *TUB* and *TUA*, *PP2A* and *TUB*, and *CYP* and *TUB*, respectively. Interestingly, all three software programs showed the worst expression stability for *HSP*.

**Table 3:** Expression stability of the 10 candidate reference genes calculated by BestKeeper analysis.

Rank	Salt treatment		Drought treatment		Heat treatment		Cold treatment		ABA treatment		Different organs		Total samples	
	Gene	CV ± SD	Gene	CV ± SD	Gene	CV ± SD	Gene	CV ± SD	Gene	CV ± SD	Gene	CV ± SD	Gene	CV ± SD
1	*ACT*	1.46 ± 0.38	*TUA*	2.10 ± 0.56	*UBC*	2.6 ± 0.74	*TUB*	3.18 ± 0.84	*PP2A*	2.48 ± 0.67	*CYP*	2.38 ± 0.70	*PP2A*	3.52 ± 0.98
2	*PP2A*	2.42 ± 0.65	*ACT*	2.63 ± 0.70	*CYP*	2.66 ± 0.77	*TUA*	3.44 ± 0.97	*TUB*	3.05 ± 0.75	*TUB*	2.52 ± 0.68	*ACT*	3.62 ± 0.96
3	*GAPDH*	4.41 ± 1.08	*TUB*	2.75 ± 0.78	*TUB*	2.72 ± 0.71	*ACT*	3.66 ± 1.02	*EF1-α*	3.13 ± 0.68	*PP2A*	2.53 ± 0.73	*TUB*	3.84 ± 1.00
4	*TUA*	4.23 ± 1.11	*PP2A*	2.94 ± 0.78	*PP2A*	2.75 ± 0.78	*PP2A*	3.62 ± 1.04	*His*	3.43 ± 0.81	*ACT*	2.61 ± 0.72	*CYP*	4.19 ± 1.20
5	*TUB*	4.34 ± 1.12	*EF1-α*	3.48 ± 0.82	*ACT*	3.17 ± 0.85	*CYP*	3.77 ± 1.11	*CYP*	3.49 ± 0.95	*TUA*	3.32 ± 0.94	*UBC*	4.48 ± 1.28
6	*UBC*	4.28 ± 1.23	*UBC*	3.37 ± 0.97	*GAPDH*	3.32 ± 0.83	*His*	5.67 ± 1.41	*ACT*	3.54 ± 0.90	*EF1-α*	4.42 ± 1.08	*TUA*	4.56 ± 1.21
7	*CYP*	4.49 ± 1.30	*GAPDH*	3.53 ± 0.87	*His*	3.67 ± 0.95	*UBC*	5.58 ± 1.57	*GAPDH*	4.15 ± 0.97	*GAPDH*	4.46 ± 1.15	*GAPDH*	4.84 ± 1.19
8	*His*	5.26 ± 1.31	*His*	4.74 ± 1.19	*TUA*	3.71 ± 0.96	*GAPDH*	5.71 ± 1.44	*UBC*	3.8 ± 1.07	*His*	4.81 ± 1.27	*EF1-α*	5.27 ± 1.24
9	*EF1-α*	5.73 ± 1.36	*CYP*	4.96 ± 1.41	*EF1-α*	4.26 ± 1.01	*EF1-α*	5.78 ± 1.38	*TUA*	4.02 ± 1.01	*HSP*	5.74 ± 1.62	*His*	5.57 ± 1.39
10	*HSP*	6.50 ± 1.88	*HSP*	5.91 ± 1.67	*HSP*	9.99 ± 2.50	*HSP*	5.74 ± 1.60	*HSP*	8.29 ± 2.21	*UBC*	5.77 ± 1.73	*HSP*	7.92 ± 2.18

### Reference gene validation

**Figure 4:**
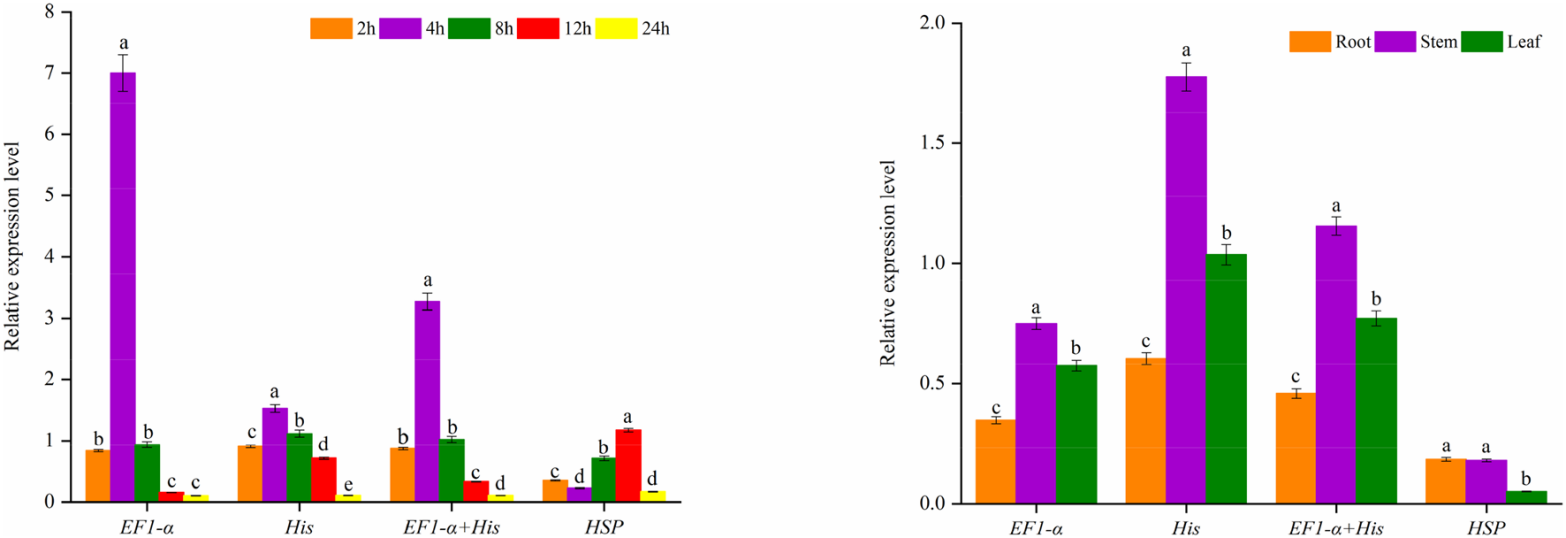
Relative expression of *NtCER7* under salt stress and in different organs of *N. tangutorum*. *EF1-α* and *His* and *EF1-α* + *His* were used as one or two of the most stable reference genes; *HSP* was used as the least stable reference gene. Different letters indicate significant differences in the expression of the target gene based on three biological replications (*P* < 0.05). The figures were generated by using OriginPro software (version 2018; https://www.originlab.com/).

In order to verify the screening results of geNorm and NormFinder, we used the two genes (*EF1-α* and *His*) with the best ranking stability and the least stable gene (*HSP*) as reference genes. We then analyzed the expression pattern of *NtCER7* related to the regulation of the cuticular wax biosynthesis of *N. tangutorum* under salt stress and in different organs by qPCR (Fig. 4). It has been found that salt stress can lead to increased waxiness around the stomata of the epidermis of *N. tangutorum*^33^. The increase in the waxiness of *N. tangutorum* is closely related to the increase in the expression levels of *NtCER7*. When *EF1-α* and *His* were used as reference genes under salt stress, the trend of *NtCER7* expression showed an inverted V shape from 2 to 24 h, with a peak at 4 h. When the data of *NtCER7* expression were normalized with these two reference gene combinations, consistent results were obtained. When using *HSP*, the results showed that the lowest value appeared at 4 h, and the trend increased from 4 to 12 h. Meanwhile, in different organs, when *EF1-α*, *His*, and *EF1-α* + *His* were used to calibrate the expression data of *NtCER7* they all showed consistent results. The expression level of *NtCER7* was greatest in the stems, followed in descending order by leaves and roots. However, the expression pattern of *NtCER7* normalized by *HSP* in different organs was highest in roots, followed by stems, and lowest in leaves. *CER7* mainly regulates cuticular wax biosynthesis in *Arabidopsis* stems^34^, so its expression level in the stems is significantly higher than that in the roots. In our results, it was clear that the expression of *NtCER7* in the stems was underestimated. Therefore, based on the above experimental results, we believe that the results of geNorm and NormFinder were reliable.

## Discussion

qPCR is a the commonly used technique for studying gene expression^35^. However, the results of quantitative experiments are easily affected by factors including the specificity of primers, the length of amplification products, and the quality of RNA samples. The most important factor is the stability of the reference gene^36^. Selecting a suitable gene as a reference is the key to studying the expression pattern of the target gene. In general, some housekeeping genes are used as reference genes, such as *ACT*, *EF1-α*, and *TUB*, which are expressed in various types of cells in an organism. The expression levels of these genes are less affected by environmental factors. However, the stability of their expression changes greatly in different species, under different abiotic stresses, and in different organs^16,21,37^. In other words, the selection of suitable reference genes under specific experimental conditions has become an important prerequisite for the qPCR process.

*N. tangutorum* is a pioneer plant for soil desertification and salinization control due to its strong resistance to stress^38^. Therefore, it is of great significance to develop and utilize the anti-stress gene resources of *N. tangutorum* and to study its molecular mechanisms. Unfortunately, due to the lack of genetic information, the research progress on the molecular mechanism of the stress resistance of *N. tangutorum* has been very slow; to date there has been no research on suitable reference genes in *N. tangutorum*. In our study, 10 typical candidate reference genes (*ACT*, *GAPDH*, *TUA*, *TUB*, *CYP*, *UBC*, *His*, *PP2A*, *HSP*, and *EF1-α*) were selected from the transcriptome database of *N. tangutorum*, and their expression stability under salt, drought, heat, cold, ABA, and in the root, stem and leaf was evaluated using geNorm, NormFinder, and BestKeeper software. As shown in the analysis results of Tables 2 and 3, Figs. 1 and 2, the optimal gene ranking order obtained by the three software programs differed under different experimental conditions. First, from the perspective of the transcriptional abundance and variation of candidate genes, *EF1-α*, *GAPDH*, *His*, *ACT*, *TUB*, and *TUA* met the requirements for ideal reference gene. geNorm analysis suggested that *EF1-α* and *His* were more suitable as reference genes among the 10 genes from all samples of *N. tangutorum*. Similar results were obtained from NormFinder analysis. However, *PP2A* and *ACT* showed better stability for use in the BestKeeper analysis in all samples. This was not surprising, because the algorithms and procedures used by the three analysis software programs are different, leading to inconsistent results. The BestKeeper algorithm provided a comprehensive analysis of the stability of the reference gene based on its SD and CV between the Cq values^31^. The geNorm and NormFinder algorithms focused more on the variation of reference genes^29,30^. This situation also occurred with geNorm and NormFinder analysis in *Pyrus pyrifolia*^39^, *dimocarpus longan*^40^, and *Miscanthus lutarioriparia*^19^. It has been reported that BestKeeper can obtain the most unstable gene stability ranking results^41^. This was consistent with the results of our experiment. Therefore, we used a previously described method^16^ to determine the best reference gene based on all three algorithms (see Supplementary Table S3). According to the frequencies of the reference genes as ranked by the three algorithms across the different treatments (salt, drought, heat, cold, ABA, organs, and total), *EF1-α* and *His* were the best reference genes, while *HSP* showed the worst stability among the 10 candidate reference genes.

To validate the accuracy of the experimental results, we used *EF1-α*, *His*, and *HSP* as reference genes and analyzed the expression patterns of *NtCER7*, a gene related to the regulation of cuticular wax biosynthesis in *N. tangutorum* under salt stress and in different organs by qPCR. The results showed that when *EF1-α*, *His*, and *EF1-α* + *His* were used to calibrate the expression of *NtCER7*, the results obtained were similar. However, when *HSP* was used to calibrate the expression data, there were serious variations (Fig. 4). Previous studies have suggested that unstable internal control genes introduce significant errors in the qPCR analysis, leading to misinterpretation of expression data^42−45^. Our results further confirmed these views. Consequently, reliable reference genes are a prerequisite for obtaining accurate results in qPCR experiments. In general, in our study *EF1-α* and *His* were found to have the best stability under different abiotic stresses and hormone stimuli and in different organs. *EF1-α* and *His* can be used as internal reference genes for *N. tangutorum* for subsequent experimental research.

## Materials and methods

### Plant materials

Seeds of *N. tangutorum*, collected from QingHai Province in Northwest China, were used in this study. The seeds were sown in a seedling-raising plate filled with clean river sand and were randomly placed in the artificial climate chamber of Gansu Agricultural University (103°70′E; 36°09′N), Lanzhou, China. The cultivation conditions were 16 h of light and 8 h of darkness, and the temperatures under light and dark conditions were 26 °C and 22 °C, respectively. The relative humidity was 60%. After germination, the seedlings were irrigated with 1/4 strength Hoagland’s solution once a week. When the seedlings had grown for two weeks, the weaker seedlings were removed. After the seedlings had grown for 4 weeks, the seedlings that displayed good, consistent growth were randomly collected for different experimental treatments. For drought and salt stress, seedlings were treated with 20% polyethylene glycol and 300 mM NaCl, respectively. For heat and cold stress, the seedlings were subjected to temperatures of 45 °C and 4 °C, respectively. For hormonal stimulus, the seedlings were sprayed with 100 µM ABA. All of the above samples were collected at 2, 4, 8, 12, and 24 h. At the same time, samples of different organs (rhizomes and leaves) of untreated seedlings were collected. Untreated seedlings in the incubator were used as blank controls. The collected samples were cleaned with purified water, frozen in liquid nitrogen and placed in an ultra-low temperature refrigerator for subsequent use. The experiment was performed with three biological replicates.

### Total RNA extraction and cDNA synthesis

Total RNA was extracted from each sample using the RNAprepPure Plant Kit DP441 (Tiangen Biotech Co., Ltd, Beijing, China) according to the manufacturer’s instructions, followed by DNase I (Tiangen Biotech Co., Ltd.) treatment to eliminate DNA contamination. The purity and concentration of RNA samples were measured using a TGem spectrophotometer (Tiangen Biotech Co., Ltd.) and integrity was checked on agarose gel electrophoresis. RNA samples with a concentration higher than 200 ng/μL and a A260/A280 ratio between 1.9 and 2.1 were required for cDNA preparation. The reverse transcription kit (TaKaRa, Shiga, Japan) was used to reverse transcribe each sample of RNA into first-strand cDNA according to the user manual. Briefly, 10 µL of Master Mix was prepared, including 1 µg RNA, 2 µL 5 × gDNA Eraser Buffer, 1 µL gDNA Eraser, and a certain amount of RNase Free dH_2_O. The Master Mix was then incubated at 42 °C for 2 min. After that, 1 µL PrimeScript RT Enzyme Mix I, 1 µL RT Primer Mix, 4 µL 5 × PrimeScript Buffer 2 (for Real Time) and 4 µL RNase Free dH_2_O were added and then incubated at 37 °C for 15 min. Finally, the RT reaction was terminated by incubating at 85 °C for 5 s. The obtained cDNA was then diluted with 10 times RNase Free dH_2_O to conduct qPCR.

### Selection of candidate reference genes and the design of qPCR primers

According to the related research literature on the plant reference gene^13–22^, we selected the conventional reference genes suitable for gene expression normalization as candidate reference genes. Their names were used to search the transcriptome database of *N. tangutorum* and the genes with transcription abundance fragments per kilobase of exon per million fragments mapped (FPKM) values > 10 were selected. The data that support the findings of this study have been deposited in the CNSA (https://db.cngb.org/cnsa/) of CNGBdb with accession number CNP0001137. Moreover, to ensure the reliability and correctness of the proposed reference gene, we compared the retrieved unigene sequences in the *Arabidopsis* protein database by BLASTX. Finally, 10 candidate reference gene sequences (*ACT*, *GAPDH*, *TUA*, *TUB*, *CYP*, *UBC*, *His*, *PP2A*, *HSP*, and *EF1-α*) with the highest homology were obtained. The annotation and comparison information of the 10 reference genes are listed in Supplementary Table S1. The quantitative primers of the candidate reference genes were designed by the Primer3Plus online website (https://www.primer3plus.com/cgi-bin/dev/primer3plus.cgi). The parameters were as follows: the length of the primers and the amplification product size were 18–27 bp and 100–200 bp, respectively, the range of melting temperature (Tm) was 65–75 °C, and the GC was 40–60%. Primers were synthesized by Sangon Biotech. (Shanghai) company. The standard curve was constructed with a ten-fold dilution of cDNA, and the amplification efficiency (*E*) and correlation coefficient (*R*^2^) of primers were calculated using the standard curve. The amplification efficiency (*E*) of each primer pair was calculated by the curve slope using *E* = [10^(−1/*slope*)^ − 1] × 100%^28^.

### qPCR analyses

The qPCR reaction was performed using a QuantStudio 5 real-time fluorescence quantitative PCR system (Applied Biosystem, CA, USA) and Hieff qPCR SYBR Green Master Mix (Yeasen Biotech, Shanghai, China). The 20 µL reaction system consisted of SYBR Green Master Mix (10 µL), both the forward and reverse primers (0.4 µL), 1 µL of the cDNA template (diluted ten-fold with RNase-free dH_2_O), and 8.2 µL of RNase-freed dH_2_O. The PCR program was as follows: first step, initial denaturation at 95 °C for 5 min; second step, amplification at 95 °C for 10 s, 60 °C for 30 s, 40 cycles; and third step, dissolution at 95 °C for 15 s, 60 °C for 1 min and 95 °C for 15 s.

### Stability analysis of candidate reference genes

The stability of candidate reference genes under a series of experimental conditions, including different organs (root, stem, and leaf), abiotic stresses (salt, drought, heat, and cold) and hormone stimuli (ABA) was evaluated using three software programs geNorm (version 3.5; https://medgen.ugent.be/genorm/), NormFinder (version 0.953; https://moma.dk/normfinder-software), and BestKeeper (version 1.0; https://www.gene-quantification.de/bestkeeper.html) with different algorithms. geNorm can calculate a parameter M that measures the stability of the reference gene expression. The M value is negatively related to the stability of the gene, that is, the smaller the M value, the higher the stability value of the gene. geNorm can also calculate the paired variation V value of the normalized factor after introducing a new internal reference gene, and the number of optimal internal reference genes can be determined according to the Vn/Vn + 1 value^29^. The principle of the NormFinder and geNorm programs is similar but the calculation method is different. NormFinder analyzes the stable value of the expression stability by calculating the variance, including intra group and inter group variance^30^. BestKeeper analyzes gene stability from the SD and CV of the reference gene Cq value. Generally, more stable genes have lower SD and CV values^31^. For geNorm and NormFinder analysis, the raw Cq values need to be converted into a relative quantities according to the formula: 2^−ΔCq^, where ΔCq is equal to the corresponding Cq value minus the minimum Cq value. BestKeeper analysis was based on the raw Cq values.

### Validation of reference genes

In order to further verify the reliability of the reference genes screened by the software, the expression pattern of *NtCER7*, a gene related to the regulation of cuticular wax biosynthesis, was analyzed under salt stress and in different organs of *N. tangutorum*. The specific primer design is detailed in Table 1. The relative expression of the target gene *NtCER7* was calculated by the 2^−ΔΔCT^ formula^32^.

### Statistical analyses

Statistical analyses were performed using SPSS 21.0 software. The figures were generated by using OriginPro software (version 2018; https://www.originlab.com/).

## Conclusion

To the best of our knowledge, this study is the first to reveal the most suitable reference genes for *N. tangutorum*. The expression stability of 10 typical candidate reference genes was evaluated by the software programs geNorm, NormFinder, and BestKeeper. We determined that *EF1-α* and *His* are the most ideal reference genes and that *HSP* showed lower expression stability under a series of experimental conditions, including in different organs (root, stem, and leaf) and under abiotic stresses (salt, drought, heat, and cold) and hormone stimuli (ABA). At the same time, the target gene *NtCER7* was used to validate the analysis results. This work will be beneficial for studying the gene expression of *N. tangutorum* and other *Nitraria* species in the future.

## Supplementary information

Supplementary Figure.

Supplementary Tables.

## Data Availability

The datasets generated and/or analyzed during the current study are available from the corresponding author on reasonable request.
