# Supplementary material for: Systematic selection and validation of suitable reference genes for quantitative real-time PCR normalization studies of gene expression in Nitraria tangutorum
Source: Sci Rep. 2020 Sep 28;10:15891. doi: 10.1038/s41598-020-73059-3 (PMC7522712; doi:10.1038/s41598-020-73059-3)
Supplement: Supplementary file 1 — Supplementary Figure. [file 41598_2020_73059_MOESM1_ESM.pdf]

Systematic selection and validation of suitable reference genes for quantitative real-time PCR normalization studies of gene expression in *Nitraria tangutorum*

Bo Wang[1], Huirong Duan[3], Peifang Chong[1], Shiping Su[1], Lishan Shan[1], Dan Yi[1], Lirong Wang[2]*, Yi Li[1]*

[1]College of Forestry, Gansu Agricultural University, Lanzhou, 730000, China. [2]The College of Ecological Environment and Resources; Institute of Ecology and Environment of Qinghai-Tibet Plateau; Key Laboratory of Biotechnology and Analysis and Test in Qinghai-Tibet Plateau; Laboratory of Resource Chemistry and Eco-environmental Protection in Tibetan Plateau, Qinghai Nationalities University, Xining, 810007, China. [3]Lanzhou Institute of Husbandry and Pharmaceutical Science, Chinese Academy of Agricultural Sciences, Lanzhou, 730000 , China.

*email: 412567406@qq.com (Associate corresponding author); liyi@gsau.edu.cn (Principal corresponding author)

ACT

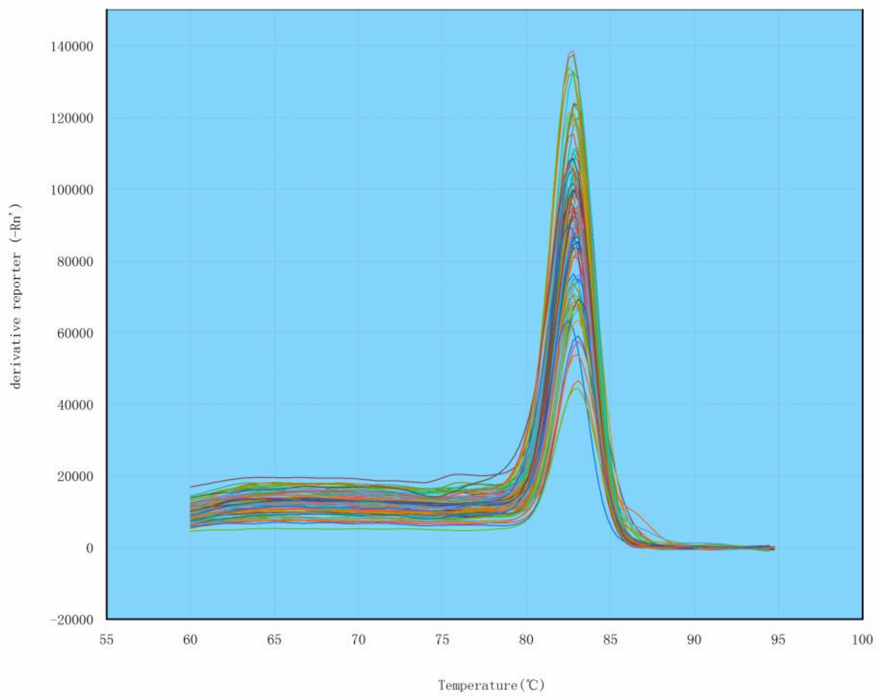

140000
120000
100000
80000
60000
40000
20000
0
−20000
derivative reporter (-Rn')
55
60
65
70
75
80
85
90
95
100
Temperature(℃)

GAPDH

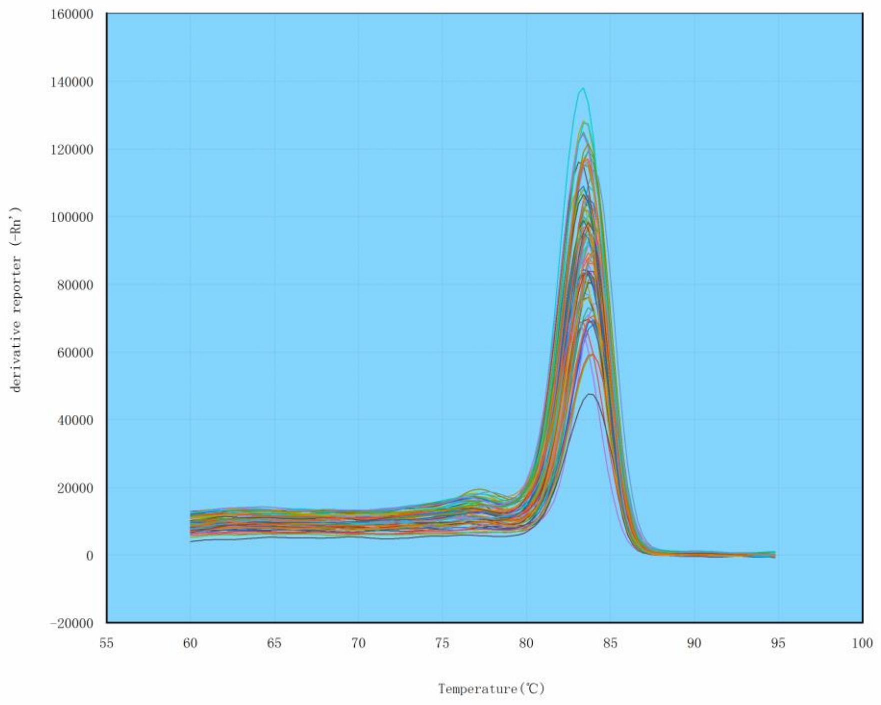

160000
140000
120000
100000
80000
60000
40000
20000
0
−20000
derivative reporter (-Rn')
55
60
65
70
75
80
85
90
95
100
Temperature(℃)

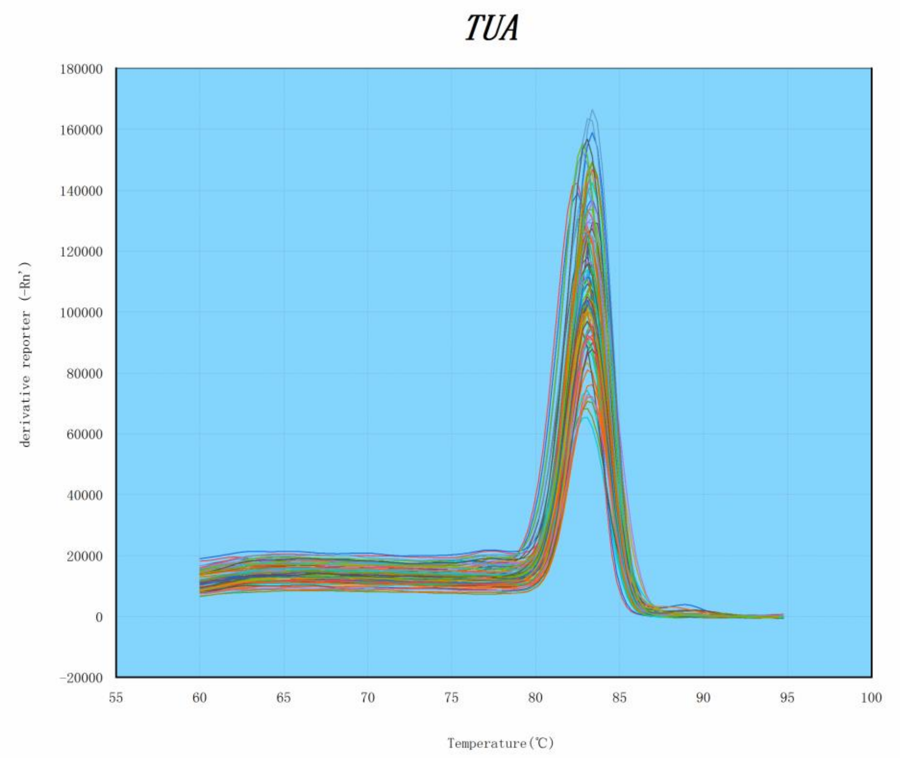
TUA
derivative reporter (-Rn')
180000
160000
140000
120000
100000
80000
60000
40000
20000
0
-20000
55
60
65
70
75
80
85
90
95
100
Temperature(℃)

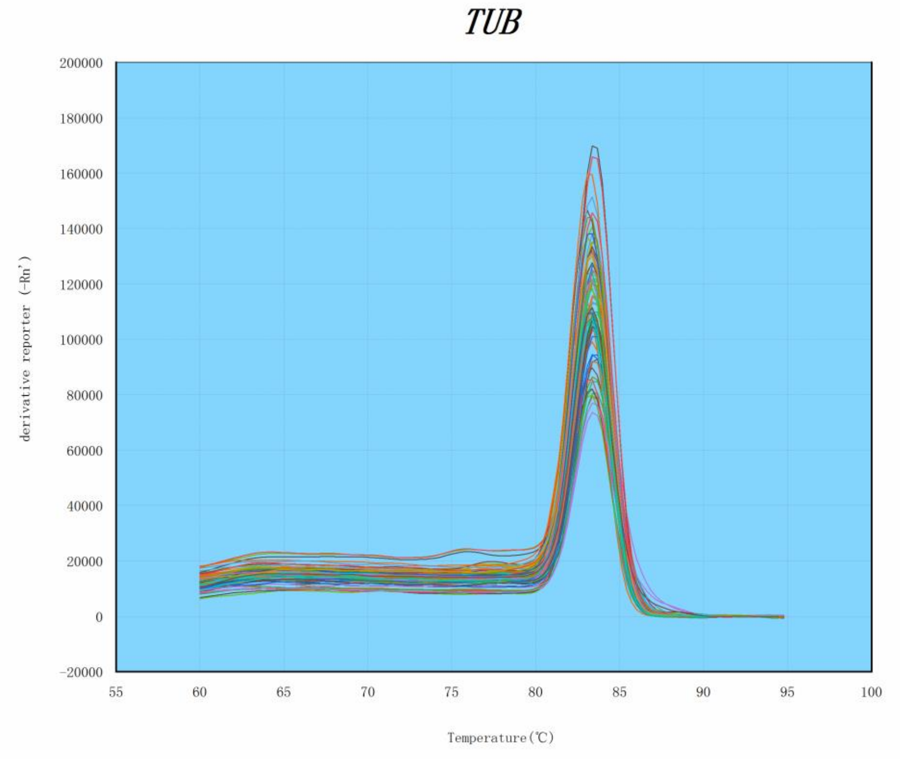
TUB
derivative reporter (-Rn')
200000
180000
160000
140000
120000
100000
80000
60000
40000
20000
0
-20000
55
60
65
70
75
80
85
90
95
100
Temperature(℃)

CYP

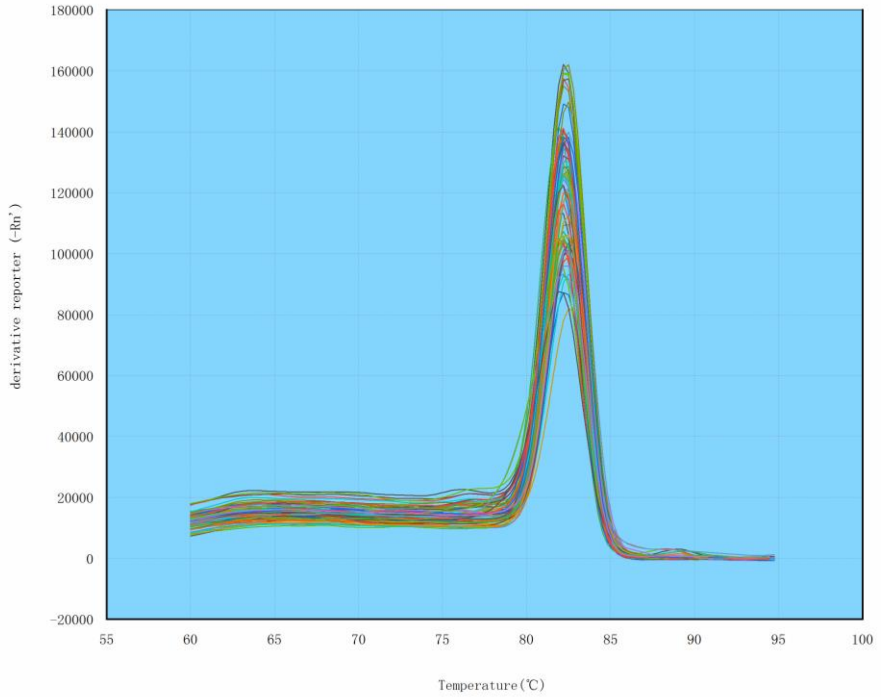
180000
160000
140000
120000
100000
80000
60000
40000
20000
0
-20000
derivative reporter (-Rn')
55
60
65
70
75
80
85
90
95
100
Temperature(℃)

UBC

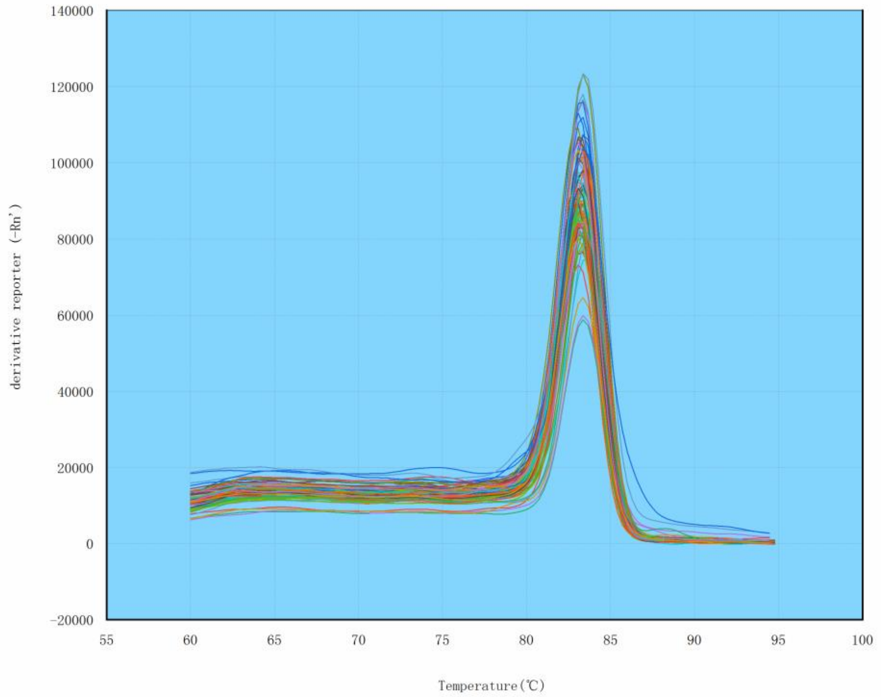
140000
120000
100000
80000
60000
40000
20000
0
-20000
derivative reporter (-Rn')
55
60
65
70
75
80
85
90
95
100
Temperature(℃)

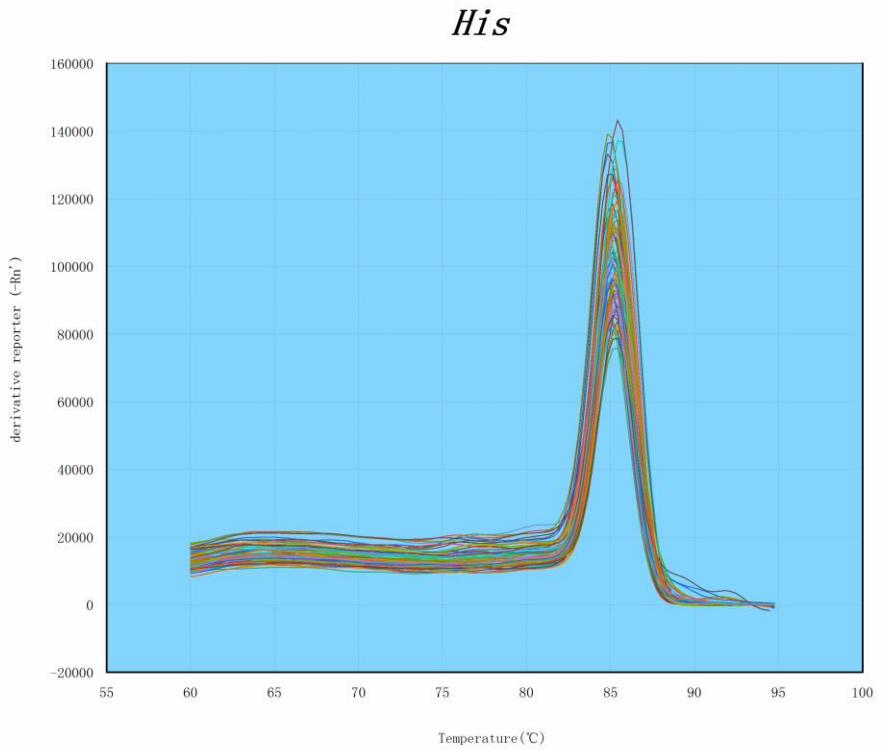

His
160000
140000
120000
100000
80000
60000
40000
20000
0
-20000
derivative reporter (-Rn')
55
60
65
70
75
80
85
90
95
100
Temperature(℃)

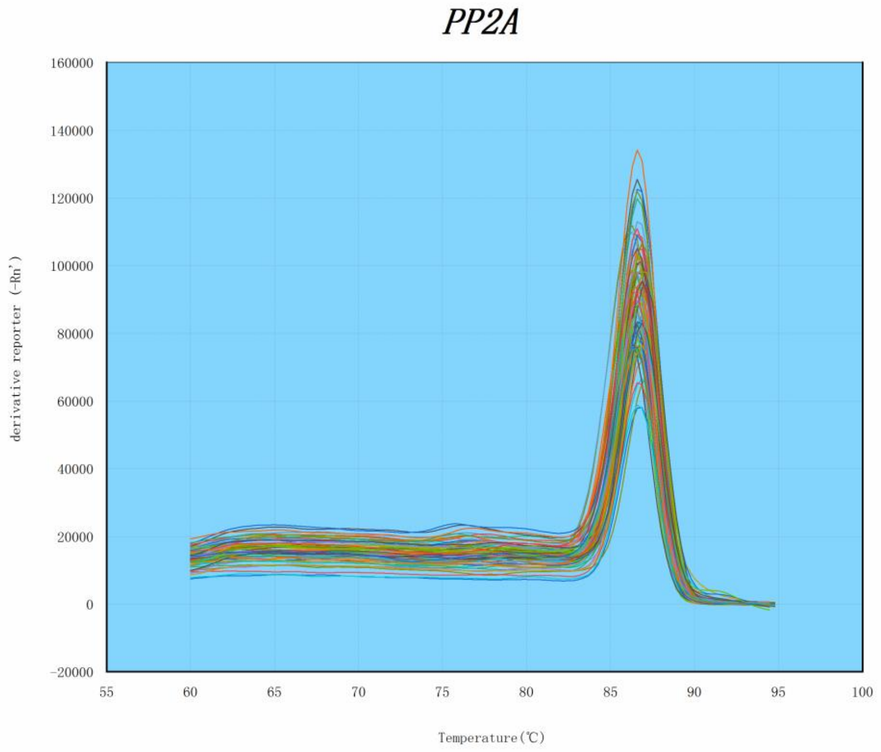

PP2A
160000
140000
120000
100000
80000
60000
40000
20000
0
-20000
derivative reporter (-Rn')
55
60
65
70
75
80
85
90
95
100
Temperature(℃)

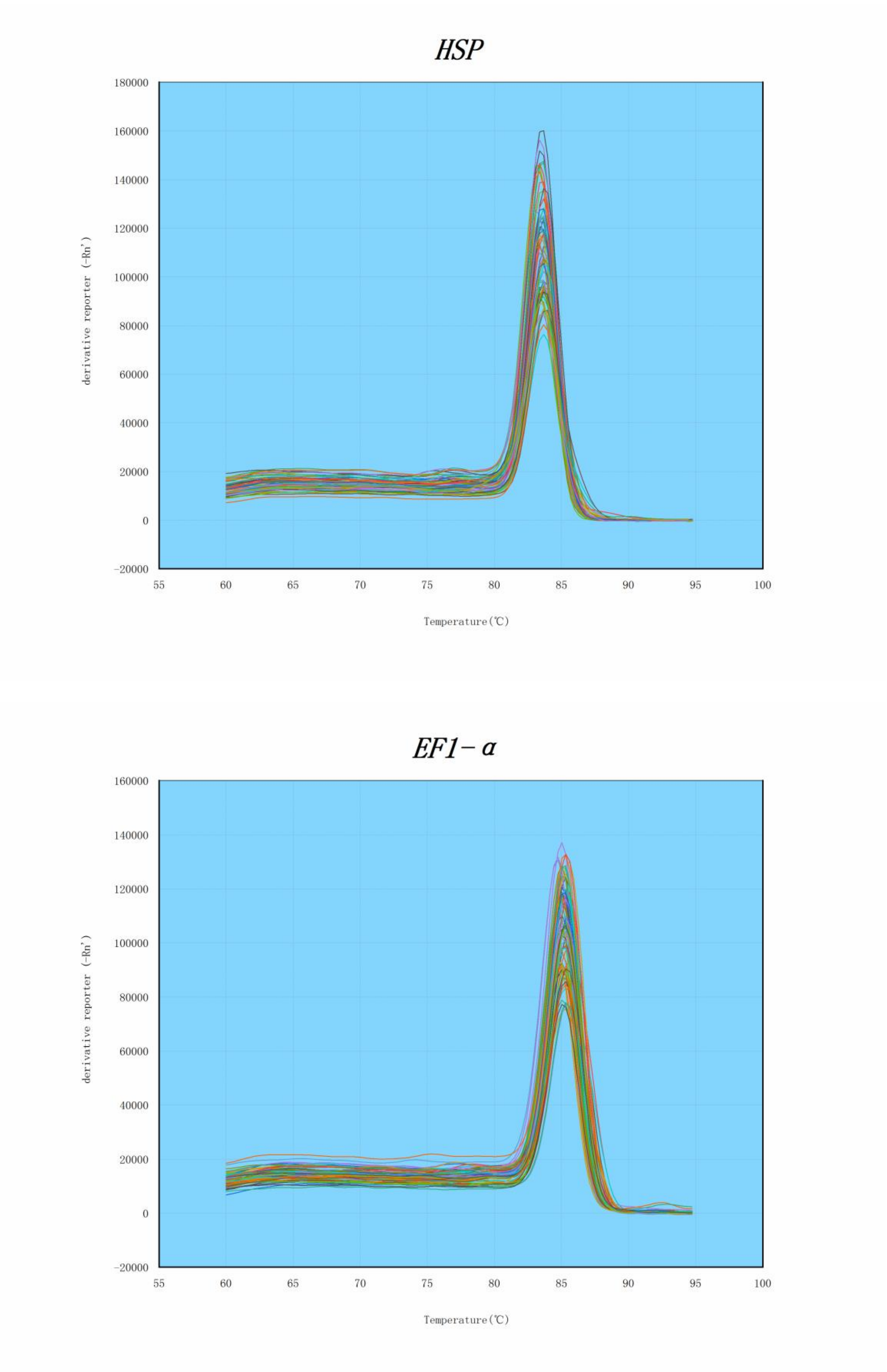

Figure S1. Melting curve of 10 candidate reference genes of *N. tangutorum*
